# Large-Area Photoreceptor Degeneration Model in Rabbits by Photocoagulation and Oxidative Stress in the Retina

**DOI:** 10.3389/fnins.2021.617175

**Published:** 2021-06-10

**Authors:** Zhexuan Wang, Chenli Feng, Ruyi Yang, Tingting Liu, Yin Chen, Aihua Chen, Biao Yan, Yuanzhi Yuan, Jiayi Zhang

**Affiliations:** ^1^State Key Laboratory of Medical Neurobiology, Department of Ophthalmology, MOE Frontiers Center for Brain Science, Zhongshan Hospital, Institutes for Brain Science, Fudan University, Shanghai, China; ^2^Department of Ophthalmology, Eye and Ent Hospital of Fudan University, Shanghai, China; ^3^Key Laboratory of Brain Functional Genomics, Primate Research Center, East China Normal University, Shanghai, China

**Keywords:** photocoagulation, light pupillary reflex, rabbit model, oxidative stress, electroretinography

## Abstract

Photocoagulation is used for the treatment of retinal ischemic disease. However, due to the invasive nature of photocoagulation and variety of melanin concentrations between individuals, it is challenging to avoid damaging the adjacent photoreceptors and inducing several side effects. Previous studies indicate the role of laser power, duration, and spot size on retinal lesions, but the effect of interspot distance of the laser pulses needs to be considered in panretinal photocoagulation. In this study, we examine different parameters of photocoagulation on lesions of the retina in rabbit, finding that the lesion level of the outer nuclear layer of the retina depended on the pulse duration and laser spot size, and decreasing interspot distance could completely abolish the photoreceptor layer. The degeneration of the photoreceptor by photocoagulation occurred in 24 h and was not restored afterward. We then conducted panretinal photocoagulation in rabbit and found that oxidative stress was decreased in the inner nuclear layer of the retina, and pupillary light reflex and ERG signals were impaired. Our study could provide a rabbit model to explore the mechanism of photoreceptor degeneration and therapies for the side effects after photocoagulation.

## Introduction

Retinal photocoagulation is considered a gold standard for the therapy of retinal ischemic disease, such as proliferative diabetic retinopathy and retinal vein occlusion (Reddy and Husain, 2018). During photocoagulation, laser light is absorbed by melanin in retinal pigment epithelium (RPE) cells and converted into heat, causing focal coagulation, necrosis, and hemostasis at RPE, Bruch’s membrane (BM), and photoreceptor cells (Lock and Fong, 2011; Querques et al., 2018). Therefore, due to the invasive nature of photocoagulation, it also induces serious side effects, including central scotoma, permanent retinal scarring, and loss of visual field and night vision (Pender et al., 1981; Fong et al., 2007). There are some hypotheses offered to explain the mechanism of laser-induced retinal damage (LIRD), including reduction in oxygen consumption, photoablative debulking of the retina by photocoagulation, and heat-shock protein (HSP) activation (Chhablani et al., 2018). A previous study shows that the RPE cell death after thermal irradiation may take time and mostly undergoes apoptosis, unless cells are immediately killed, but the cellular responses and therapeutic mechanisms are still unclear (Kern et al., 2018).

To minimize the side effects of photocoagulation, selective retinal therapy (SRT) was applied as a new therapeutic laser procedure for retinal diseases (Chhablani et al., 2018). SRT selectively targets RPEs and avoids thermal damage of the adjacent photoreceptors and choriocapillaris, which causes a high peak temperatures around the melanosomes and a low sublethal temperature increase in the adjacent tissue structures (Framme et al., 2004). However, because the melanin concentrations are different among patients or even in regions within an eye and the lesions in RPE are invisible through an ophthalmoscope (Weiter et al., 1986), localized SRT without excessive burning and collateral damage is still challenging. Previous studies indicate the role of laser power, duration, and spot size on retinal lesions (Jain et al., 2008), but during large-area photocoagulation, the effect of the interspot distance of the laser pulses needs to be considered. Meanwhile, the mechanism underlying photoreceptor degeneration after photocoagulation remains unclear. A proper animal model for studying cellular mechanisms would be helpful for further eliminating side effects by SRT.

Due to the convenience of generating transgenic animals, rodents seem to be a good animal model for studying the molecular and cellular mechanisms of photoreceptor degeneration by photocoagulation. However, the anatomical structure and size of human and rodent eyes are significantly different, preventing rodent models from further contributing to translational studies. The anatomy of eyes in nonhuman primates are very similar to that in humans, in particular, the existence of a macular structure, and translational studies often use nonhuman primates for electrophysiological and behavioral experiments (Nishida et al., 2010; Pennesi et al., 2012; Shirai et al., 2016). However, nonhuman primates are expensive and have a long breeding cycle. Despite the lack of macula, the size of eyeballs in rabbits is similar to that of humans (Kondo et al., 2009; Amirpour et al., 2012; Isago et al., 2013; Petrus-Reurer et al., 2018). The surgical tools for human patients in ophthalmology can be used directly in rabbit surgery (Petrus-Reurer et al., 2018), and fundus imaging and optical coherence tomography (OCT) for humans also enable monitoring of the rabbit retina over time (Plaza Reyes et al., 2016; Petrus-Reurer et al., 2017). Furthermore, rabbits are easy to breed and could be a good animal model in studying histological changes and mechanisms after photocoagulation.

In this study, we conducted different parameters of photocoagulation, including duration, spot size, and interspot distance of the laser pulses, and examined retinal lesions by histological approaches, electroretinography, and pupillary light reflex. We also examined the oxidative stress in the retina after photocoagulation at different time points. Our study provides a rabbit model to explore new mechanisms and therapies for the side effects after photocoagulation.

## Results

### Visualization of Whitening Level and Lesioned Spot Size by Photocoagulation in Fundus Images

**FIGURE 1:**
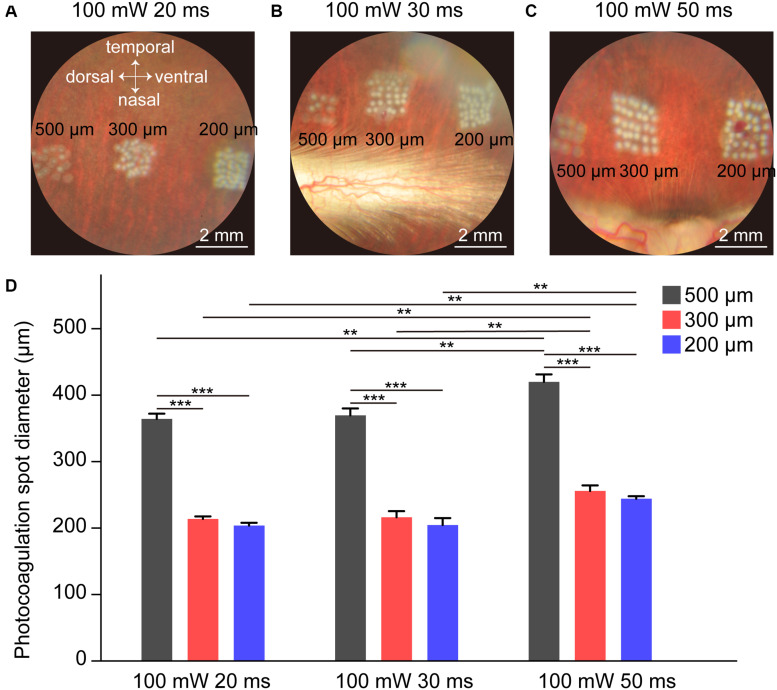
Fundus images of rabbit retina under different photocoagulation conditions 1 h after surgery. **(A–C)** Fundus images of retina after photocoagulation of 200, 300, and 500 μm laser spot size produced by 100 mW laser power and 20 **(A)**, 30 **(B)**, or 50 ms **(C)** pulse duration. Scale bar = 2 mm. **(D)** Lesion diameter on retina by photocoagulation with 200, 300, or 500 μm laser spot size produced by 100 mW laser power and 20, 30, or 50 ms duration (number of spots: n_20 ms/500 μ m_ = 9, n_20 ms/300 μ m_ = 15, n_20 ms/200 μ m_ = 15, n_30 ms/500 μ m_ = 10, n_30 ms/300 μ m_ = 15, n_30 ms/200 μ m_ = 15, n_50 ms/500 μ m_ = 9, n_50 ms/300 μ m_ = 15, n_50 ms/200 μ m_ = 14, Each set of spots comes from one retina). Data were presented as Mean ± SEM. **P < 0.01, ***P < 0.001.

The lesion level of the retina in photocoagulation experiments depends on the laser power, pulse duration, spot size, and interspot distances. Previous studies indicate that 100 mW laser power causes retinal whitening but not a ring of edema in rabbits, correlating mainly with damage on the photoreceptors (Jain et al., 2008), so we used 100 mW laser power throughout the experiments. Each laser pulse resulted in a visible bright-color lesioned spot in the fundus images. Retinal whitening level and ring of edema size indicate the intensity of the lesion; increased retinal whitening and a larger ring of edema correspond to a more severe lesion (Jain et al., 2008). As shown in Figure 1A, the whitening level of the lesioned spot in the fundus images decreased as the laser spot size increased, indicating that the level of lesion decreased as the size of the laser spot increased. Similar results were obtained in other conditions with different pulse durations (30 ms in Figure 1B and 50 ms in Figure 1C). The size of the lesioned spot on the retina increased with increasing pulse duration for 200, 300, and 500 μm laser spots (Figures 1A–D). These results indicate that the whitening level and lesioned spot size on retinal by photocoagulation was inversely proportional to the size of the laser spot and proportional to the duration of the laser pulse.

### The Degree of Retinal Damage Is Proportional to the Duration and Inversely Proportional to the Size of the Laser Spot

**FIGURE 2:**
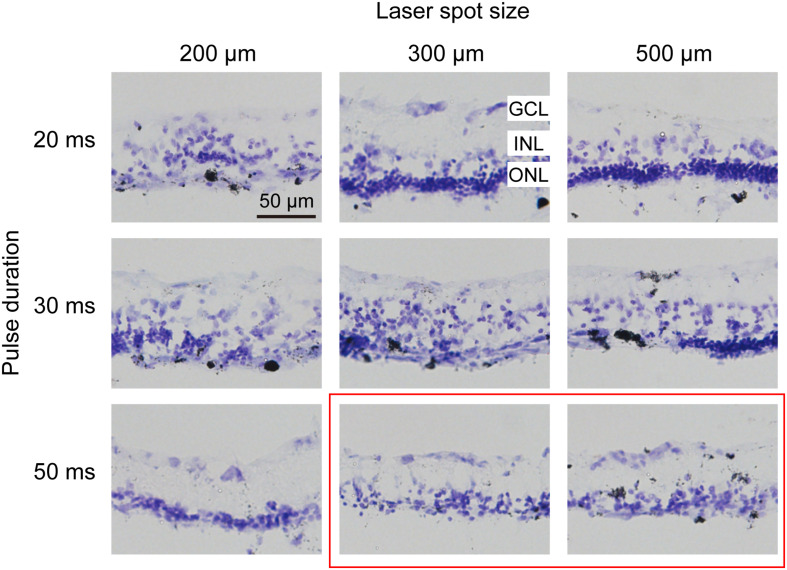
Histomorphology of rabbit retinas 7 days after photocoagulation with 100 mW laser power. Nissl staining of rabbit retina with different pulse durations and laser spot sizes under 100 mW laser power. Each column corresponds to a constant laser spot size, and each row corresponds to a constant pulse duration. The better conditions are circled by the red box. GCL, ganglion cell layer; INL, inner nuclear layer; ONL, outer nuclear layer. Scale bar = 50 μm.

To confirm which parameters of photocoagulation induce photoreceptor degeneration, which means the lesioning of the outer nuclear layer (ONL) but not the inner nuclear layer (INL) or ganglion cell layer (GCL), we examined cross-sections of the retina 7 days after photocoagulation using Nissl staining (Figure 2). We found that a 200-μm, 20-ms-duration laser spot could induce severe damage of the ONL of the retina and disarrange the structure of the retina. However, when the diameter of the laser spot was 300 μm or 500 μm (100 mW, 20 ms duration), the ONL of the retina was scarcely damaged, and a 200-μm, 30-ms laser spot disrupted the layered structure of the retina, but a 300-μm, 30-ms or 500-μm, 30-ms laser spot only partially damaged the ONL, indicating that these conditions are not efficient for lesioning the entire ONL. Also, a 200-μm, 50-ms laser spot partially damaged the INL of the retina, suggesting that the retinal tissue was over-lesioned. However, the ONL of the retina was eliminated at 300 μm and 500 μm spots (100 mW, 50 ms) without INL damage. These results suggest that the lesion level of the ONL of the retina depends on the pulse duration and laser spot size, and 50 ms, 300 μm and 500 μm laser spots seem to damage the ONL completely but not the INL of the retina.

### Spatial Distance Between Laser Spots Affected Retinal Damage Level

**FIGURE 3:**
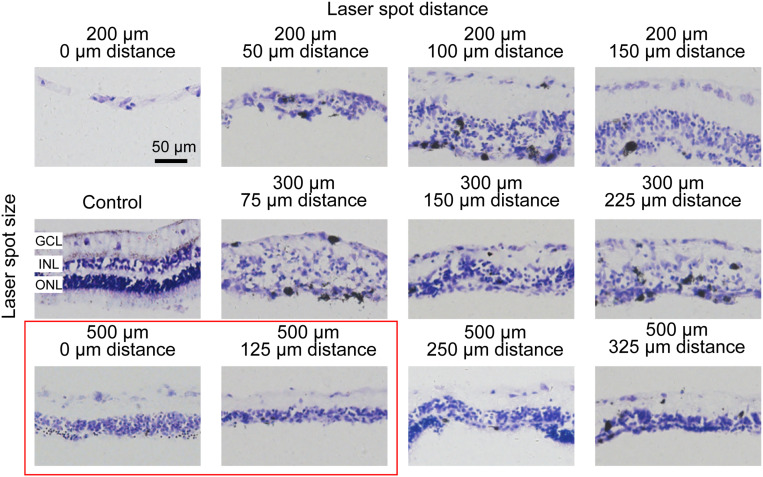
Histomorphology of rabbit retinas 7 days after photocoagulation with 100 mW laser power and 50 ms pulse duration. Histologic images for different laser spot size and distance. The control picture is in the first column and second row. Except for the control picture, each row corresponds to a constant laser spot size, and each column to different laser spot distance. The better conditions are circled by the red box. GCL, ganglion cell layer; INL, inner nuclear layer; ONL, outer nuclear layer. Scale bar = 50 μm.

To examine the photoreceptor degeneration level in the retina by large-area photocoagulation, we used laser spot arrays. Because the magnification of the rabbit eye was 0.66, the size of the laser spot at the retinal plane is different from the sizes of the lesion spots both from our own observation and in the literature (Blumenkranz et al., 2006; Framme et al., 2007). Hence, we next explored how the distance between laser spots affects retinal damage. In Figure 3, we conducted histologic analysis 7 days after the photocoagulation surgery using 100-mW laser spots with 50 ms (as used in Figure 2). In the first row of Figure 3, a 200-μm laser spot with a 0 or 50-μm interspot distance caused severe damage in the INL, ONL, and even GCL of the retina. However, when the interspot distances were increased to 100 or 150 μm, the ONL of the retina was not completely abolished. To optimize the lesion condition, we increased the spot diameter to 300 μm (50 ms duration). In the second row in Figure 3, photocoagulation with a 75-μm inter-spot distance led to the disruption of the layered structure in the INL. The level of lesions in the retina decreased with 150- and 225-μm inter-spot distances, but the effect of retinal damage is unstable. When the diameter of the laser spot increased to 500 μm with a 250- or 325-μm interspot distance, the ONL of the retina was not completely eliminated. Laser spots with 500 μm diameter and 0- or 125-μm inter-spot distance completely abolished the photoreceptor layer with intact INL and GCL.

### Long-Term Elimination of Photoreceptor Layer by Photocoagulation in Rabbit Retina

**FIGURE 4:**
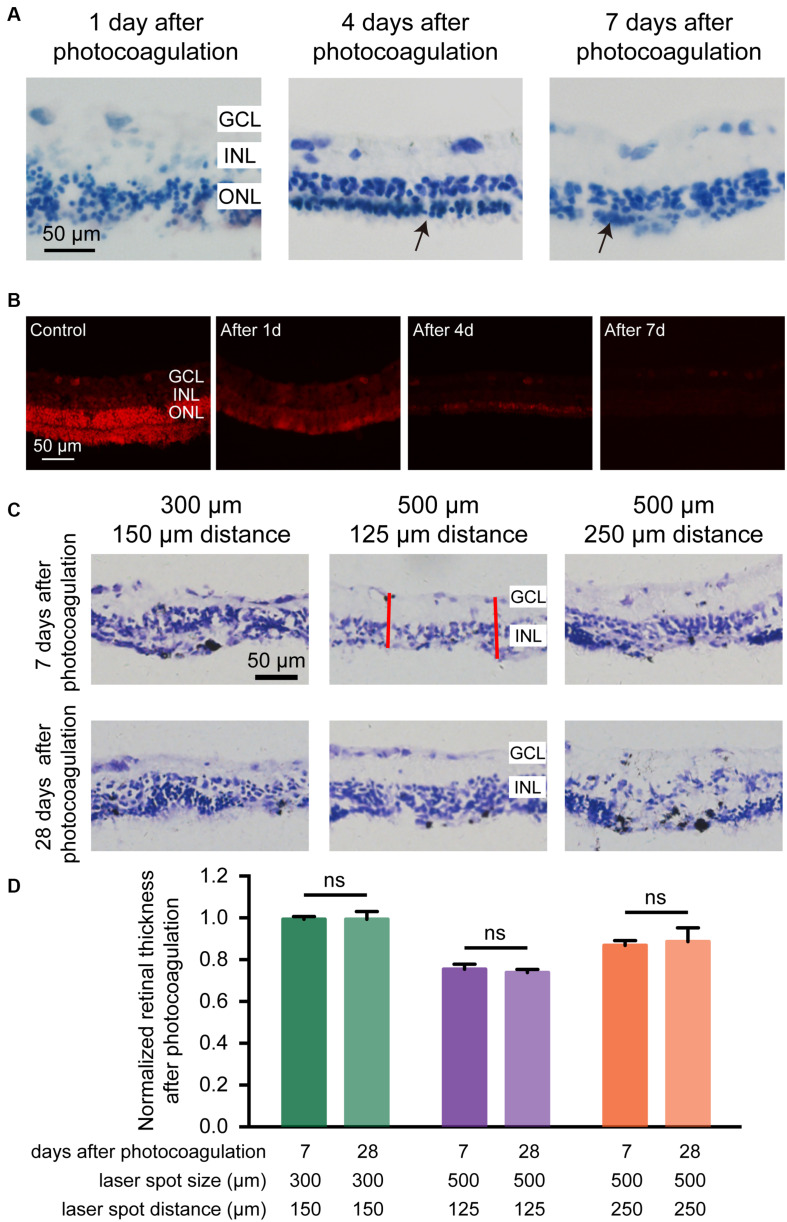
Histomorphology of rabbit retinas and normalized retinal thickness after photocoagulation surgery. **(A)** Histologic images at 1, 4, and 7 days after photocoagulation with 100 mW laser power and 50 ms pulse duration. Histologic images for 500 μm laser spot size and 0 μm laser spot distance. The arrow points to the remnant cells of the ONL. Scale bar = 50 μm. **(B)** DHE staining of control retinal sections and local damage retinal sections at 1, 4, and 7 days after photocoagulation with 100 mW laser power and 50 ms pulse duration. Stained images for 500 μm laser spot size and 0 μm laser spot distance. Scale bar = 50 μm. GCL, ganglion cell layer; INL, inner nuclear layer; ONL, outer nuclear layer. **(C)** Histologic sections of rabbit retinas 7 and 28 days after photocoagulation with 100 mW laser power and 50 ms pulse duration. Histologic images for 300/150, 500/125, and 500/250 μm laser spot size/distance. GCL, ganglion cell layer; INL, inner nuclear layer. Scale bar = 50 μm. The red line represents two locations randomly taken in each section for measuring the thickness of the retina after photocoagulation in the photocoagulation area. **(D)** Normalized photocoagulation retinal thickness in different sections at 7 and 28 days after photocoagulation under the same conditions as in **(A)**. Normalized retinal thickness is the ratio between retinal thickness in photocoagulation area and retinal thickness in nonphotocoagulation area. (7 days after photocoagulation: n_300/150 μ m_ = 8 slices, n_500/125 μ m_ = 8 slices, n_500/250 μ m_ = 6 slices; 28 days after photocoagulation: n_300/150 μ m_ = 5 slices, n_500/125 μ m_ = 6 slices, n_500/250 μ m_ = 4 slices). Data were presented as Mean ± SEM.

We examined the retinal structure and cellular morphological changes after photocoagulation, and found that 1 day after photocoagulation, the photoreceptor of the retina was almost eliminated and the ONL exhibited sparse arrangement. Seven days after photocoagulation, photoreceptor cells were completely eliminated and ONL cells were intact (Figure 4A). These data suggest that the degeneration of photoreceptor cells occurred within 24 h after photocoagulation with a few cell remnants and degenerated completely 7 days after photocoagulation. We also used DHE staining to evaluate the level of oxidative stress in retina. For the control group, the DHE fluorescence could be detected in the ONL, GCL, and photoreceptor layer. But the DHE fluorescence appeared decreased 1, 4, and 7 days after photocoagulation (Figure 4B), which was similar to the phenomena observed in a previous study (Saenz-de-Viteri et al., 2014). This might be due to the destruction of the mitochondria-rich photoreceptors by the laser treatment causing a decrease in oxygen consumption in the outer retina and allowing oxygen to diffuse from the choroidal circulation to the inner retina, decreasing oxidative stress in the ONL and INL.

We next examined the long-term effects of photocoagulation in the retina. We found that, in the 300-μm diameter/150-μm interspot distance group (100 mW power and 50 ms duration), the thickness of the retinas did not show significant differences between days 7 and 28, and part of the ONL can be observed 28 days after photocoagulation, indicating photoreceptors were not completely removed. In the 500-μm diameter/125-μm interspot distance group, the thicknesses of the retina were similar between days 7 and 28, and both showed abolished ONL and intact INL and GCL. In the 500-μm diameter/250-μm interspot distance group, there were still residual photoreceptors in the ONL on both days 7 and 28 (Figures 4C,D). These data suggest that laser pulses with 500-μm diameter and 125-μm interspot distance (100 mW power and 50 ms duration) induced stable photoreceptor degeneration.

### Panretinal Photocoagulation in Rabbit Retina With Optimal Parameters

**FIGURE 5:**
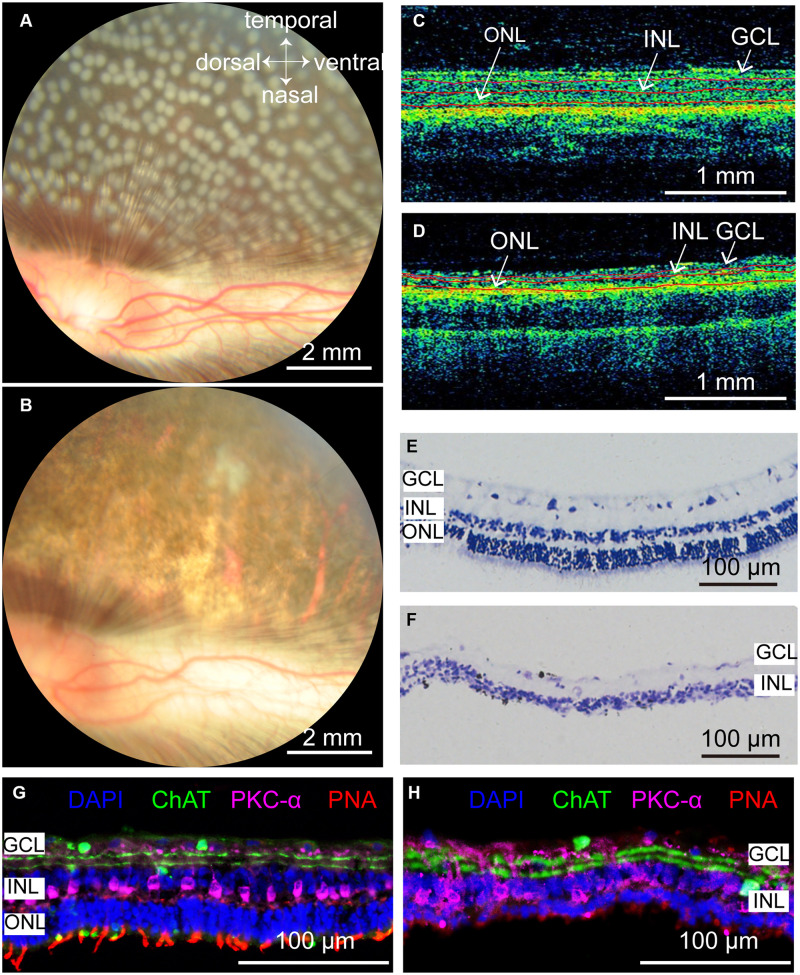
Rabbit retinal photoreceptors across the entire retina were damaged by photocoagulation with 100 mW laser power, 50 ms pulse duration, and 500 μm laser spot size. **(A,B)** Fundus photographs of extensive damage of retinal photoreceptors at 1 h and 7 days after photocoagulation. Scale bar = 2 mm. **(C)** Control OCT image of rabbit retina. Scale bar = 1 mm. **(D)** Example of OCT image of rabbit retina at 7 days after photocoagulation. Scale bar = 1 mm. Red lines in **(C,D)** mark retina stratification. **(E,F)** Histomorphology of control rabbit retina **(E)** and rabbit retina 7 days after photocoagulation **(F)**. Scale bar = 100 μm. **(G,H)** Immunofluorescence staining of control rabbit retina **(G)** and rabbit retina 7 days after photocoagulation **(H)** with cone outer segments marker PNA, bipolar cell marker PKC-α and amacrine cell marker ChAT 7 days after photocoagulation. Scale bar = 100 μm. GCL, ganglion cell layer; INL, inner nuclear layer; ONL, outer nuclear layer.

According to the results in Figure 3, the optimal parameters for selective damage of the ONL in the rabbit retina were 100 mW, 50 ms pulse duration, 500 μm diameter and 0–125 μm interspot distance. We further conducted photocoagulation on the entire rabbit retina using these parameters. Fundus images showed that the lesioned spots were all connected to each other 7 days after photocoagulation (Figures 5A,B). OCT images showed that the signals from the ONL of the rabbit retina were disturbed, but the signal of the INL and the GCL were relatively clear on day 7, indicating that most of the ONL was damaged (Figures 5C,D). As expected, Nissl staining showed that the ONL was almost abolished 7 days after photocoagulation (Figures 5E,F). The expression of cone outer segment marker PNA could not be observed in the photocoagulated retina, but PKC-α (bipolar cell marker) and ChAT (amacrine cell marker) immunochemistry signals were visible (Figures 5G,H). These results show that photocoagulation could induce selective elimination of photoreceptors over a large area.

### Disrupted Pupillary Light Reflex and ERG Recording After Panretinal Photocoagulation

**FIGURE 6:**
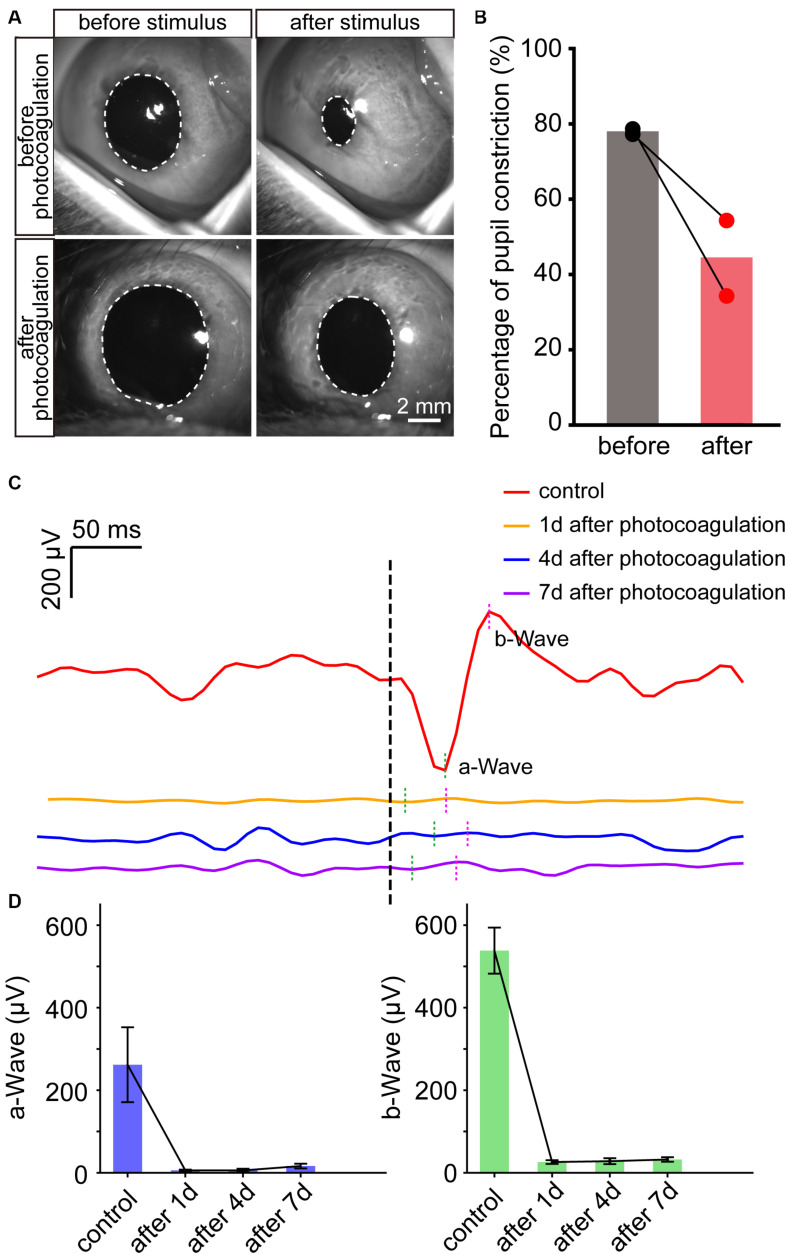
The change of pupillary light reflex and ERG in rabbits before and after photocoagulation. **(A)** The example images of pupillary direct light reflex before (top row) and 3 days after photocoagulation (bottom row) of the same rabbit eye. The position of the white dotted circle is the pupil position. Scale bar = 2 mm. **(B)** Pupil constriction ratio before and 3 days after photocoagulation of rabbits. The pupil constriction ratio is calculated as (S_0_-S_min_) / S_0_ × 100%, S_min_ is the minimum pupil area under light stimulation, and S_0_ is the pupil area of the frame before light is given. **(C)** Sample waveforms of ERG before photocoagulation, 1, 4, and 7 days after photocoagulation. **(D)** Comparison of a- and b-wave amplitudes in eyes before photocoagulation and 1, 4, and 7 days after photocoagulation.

Finally, we examined the pupillary light reflex before and after lesions of panretinal photoreceptors by photocoagulation surgery. The pupil constriction ratio was reduced significantly after photocoagulation, indicating that the photoreceptor damage caused the decrease of light response (Figures 6A,B). To evaluate the function of retinal neurons after photocoagulation, we performed electroretinogram (ERG) recording at 1, 4, and 7 days after photocoagulation. In control eyes, we could record ERG signals, and the amplitudes of the a-wave has mean values of 261.76 ± 90.73 μV while the b-wave is 538.28 ± 55.71 μV. After photocoagulation, the amplitude of a- and b-waves significantly decreased 1 day after photocoagulation, and the patterns and amplitudes did not show significant changes from days 1 to 7 after photocoagulation (Figures 6C,D). These data suggest that the RPE-photoreceptor complex function of the rabbits was obviously damaged on the first day after photocoagulation, and the effect of photocoagulation with the parameters we used may be persistent and stable.

## Discussion

In this study, we examined different parameters of photocoagulation in rabbits, which can serve as an effective large animal model for studying cellular mechanisms that come into the retina after photoreceptor degeneration. Prior to the photocoagulation surgery, the laser power and pulse duration need to be calibrated to avoid fundus bleeding due to vascular rupture during the surgery. Consistent with previous results (Jain et al., 2008), the diameter of the lesion spot increases as the pulse duration increases. Jain et al. (2008) report that the diameter of the fundus image of the lesion is larger than the spot size of the laser beam at longer pulse durations.

The level of retinal damage in different rabbit strains could also vary with the same photocoagulation parameters. The most widely used rabbits are pigmented rabbits. McHugh et al. (1995) demonstrate that photocoagulation damage in pigmented rabbits is mainly caused by the absorption of laser energy by melanin in retinal pigment epithelium and choroidal melanocytes, and retinal damage by photocoagulation in albino rabbits is induced by multiple scattering together with absorption within hemoglobin and tissue water. Under the same photocoagulation condition, chorioretinal coagulation in albino rabbits was weaker than that in pigmented rabbits (McHugh et al., 1995). Longer duration and higher power were required to achieve the same coagulation effects in albino rabbits compared with pigmented rabbits (Obana and Miki, 1989). Therefore, the lesion threshold by photocoagulation was lower in pigmented rabbits than in albino rabbits. Moreover, intravenous dye injection, such as indocyanine green (ICG), can enhance the retinal damage level by photocoagulation in rabbits (Suh et al., 1991; Matsumoto et al., 1992). The albino rabbits need less time for recovery of the intraocular pressure after photocoagulation compared with pigmented rabbits (Schubert and Federman, 1989). Therefore, it is necessary to adjust the laser power, pulse duration, and spot size according to the rabbit breeds to achieve the ideal photocoagulation effects. Moreover, pigmented rabbits could effectively absorb laser energy and cause retinal damage, which is more suitable for establishing a rabbit model of photoreceptor damage.

The effectiveness of photocoagulation also depends on the age and metabolic state. Previous studies show that, with increasing age, RPE cells thicken and become heavily loaded with metabolic fatty products (Schraermeyer and Heimann, 1999), and the content of soluble melanin in the pigment epithelium declined with age from 95 μg/mg in the 14–50 year age group to 22 μg/mg dry weight in the over 70 year age group (Schmidt and Peisch, 1986). However, the contents of melanin did not show significant differences between males and females in blue and brown eyes (Menon et al., 1992). Photocoagulation is widely used in the treatment of proliferative diabetic retinopathy. ROS was increased in the retina in diabetic mice compared with control mice, indicating that damage of the retina by photocoagulation might be more severe (Sasaki et al., 2010), and the proliferation and hexagonality of regenerating RPE cells were impaired after photocoagulation, and the regenerated RPE cells lost their original properties in diabetic mice compared with wild-type mice (Jang et al., 2019). Laser-induced choroidal neovascularization was reduced significantly in the laser-injured diabetic mice compared with the laser-injured control mice (Liu et al., 2018).

The model established by photocoagulation also has some limitations. First, this model of retinal damage is suitable for pigmented animals because laser light is mainly absorbed by melanin. The photoreceptor degeneration model established by photocoagulation is due to the trauma caused by laser impact, which is different from the progressive, hereditary pathological characteristics of RP and AMD as a result of genetic, environmental, or age-related degeneration. Moreover, the rods gradually die after progressive atrophy in RP patients, which then leads to the death of the cones, but photocoagulation causes the death of the rods and cones simultaneously, and photocoagulation-induced retinal degeneration occurs almost quickly, which is different from the progressive loss of photoreceptors in retinal degenerative diseases. Due to these limitations of the photoreceptor degeneration model by photocoagulation, the model cannot be used to study the disease progression of typical retinal degenerative diseases, nor is it suitable for studying the treatment of gene therapy, drugs, and chronic nutrition. Nevertheless, this model still has certain actual uses. The model can be used to study the effectiveness of retinal prosthesis and stem cell therapy in photoreceptor degeneration disease, and it may also be used to study the mechanism of cell death caused by oxidative stress.

In transgenic rabbit models of photoreceptor degeneration, the thickness of the ONL of the transgenic rabbit started to decrease at 2 weeks of age. By 48 weeks of age, there was still a little residual ONL of the transgenic rabbit retina. Moreover, 12 out of 80 newborn rabbits are transgenic, and 10 out of 12 survive (Kondo et al., 2009). Hence, transgenic rabbit models are slow and costly. Ahn et al. (2019) establish a local retinal degeneration rabbit model by intravitreal injection of N-methyl-N-nitrosourea (MNU). In the high-dose injection group, loss of the photoreceptor layer occurred 1 month after the injection. However, without vitrectomy, the degree of retinal degeneration is unpredictable. In addition, vitrectomy causes around 30% incidence of cataract, further reducing the success rate. Intravenous injection of IAA in rabbits induced damage in the outer but not the inner segment of the photoreceptors (Yamauchi et al., 2011). In addition, the degree of degeneration was different among animals with the same IAA dose (Liang et al., 2008). In the current study, we demonstrate that the photoreceptor degeneration model established by photocoagulation can stabilize 7 days after surgery, requires no further invasive operations, and the area of degeneration can be precisely controlled.

In summary, we develop and evaluate a reproducible and low-cost photoreceptor degeneration rabbit model by laser photocoagulation, in which selective damage was made to retinal photoreceptors within 7 days. This model can be used to induce local or large-area photoreceptor lesions. Our studies shed light on a convenient model to test potential therapies and mechanisms of cell death for photoreceptor degeneration prior to nonhuman primate studies.

## Materials and Methods

### Animals

A total of 20 healthy male adult Chinchilla Bastard rabbits were used in this study. The rabbits were obtained from Shanghai Songlian Laboratory Animal Co., Ltd. They were housed with a 12-/12-h light/dark cycle, and food and water were available ad libitum. The body weight of a rabbit is between 2.5 and 3.0 kg. All procedures were performed in accordance with the National Institutes of Health Guide for Care and Use of Laboratory Animals and were approved by Animal Care and Use Committee of Shanghai Medical College of Fudan University.

### Photocoagulation

Rabbits were anesthetized by a mix of 3% isoflurane (RWD Life Science Co., Shenzhen, China) and oxygen in a gas chamber via a custom-made mask. The position of the rabbit was kept by a custom-made body support during surgery. Before the surgery, the rabbit pupil was fully dilated by two drops of 0.5% phenylephrine hydrochloride and 0.5% tropicamide ophthalmic solution for 20 min. All laser spots were delivered by VISULAS 532s (Carl Zeiss, Dublin, CA, United States) (laser power, 100–200 mW; pulse duration, 20–200 ms; laser spot diameter, 200–500 μm) and focused on the rabbit fundus by a contact lens (Ocular Mainster Focal/Grid Laser Lens, OMRA-S-2).

### Fundus Photography and Optical Coherence Tomography

Color fundus photography (CFP) was obtained 1 h and 7 days after laser treatment to evaluate the effect of photocoagulation.

Optical coherence tomography images were obtained before and 7 days after laser treatment to evaluate changes in retinal structure with the Cirrus HD-OCT 4000 (Carl Zeiss Meditec, Inc., Dublin, CA, United States).

### Retinal Histology

Rabbits were sacrificed with a lethal dose of sodium pentobarbital under deep anesthesia, and the eyeballs were enucleated afterward. The eye was dissected in Ringer’s solution to keep cell viability. Retinal samples for DHE staining were incubated with DHE solution (5 μM) in a light-protected chamber at 37°C for 40 min and immersed for 5 min in 4% paraformaldehyde. Retinal samples for Nissl staining and immunostaining were immersed for 5 min in 4% paraformaldehyde. Then, the retina was dehydrated in graded sucrose solution and embedded in OCT compound (Sakura Finetek, United States). Retinas were sectioned into 14-μm-thick sections.

For immunohistochemistry study, slides were washed three times with 0.05 M tris buffer saline (TBS) for 15 min. After immersing slices in 0.5% Triton-X-100 for 20 min, the slides were incubated in a 10% Donkey serum (Jackson Immunoresearch, United States) blocking solution, with 1% bovine serum albumin (BSA) and 0.05% Triton-X-100. After being incubated with primary antibody (anti-choline acetyltransferase antibody, MILLIPORE (AB144P), 1:200; PNA, Vector (RL1072), 1:500; PKC alpha Monoclonal Antibody, ThermoFisher (MA1-157), 1:100) for 20 h at 4°C, the slides were washed four times for 15 min in 0.05 M TBS and incubated with secondary antibody for 1.5 h at room temperature. After washing the secondary antibody (Donkey anti-Goat conjugated to Alexa Flour 488, 1:200, Jackson ImmunoResearch, United States; Donkey anti-Mouse conjugated to Alexa Flour 647, 1:200, Jackson ImmunoResearch, United States) away with TBS, the slides were covered by 1:3000 DAPI (Sigma, United States) solution for 3 min and washed three times for 10 min with TBS. Finally, the slides were air-dried and cover-slipped.

For Nissl staining, sections washed twice for 2 min by double distilled water and then stained in 0.1 % cresyl violet solution, which was preheated to 37°C for 15 min. After that, the sides were washed in distilled water and differentiated in 30%, 70%, 95%, and absolute ethanol for 30 s, respectively. Finally, the slides were put in Xylol for 30 s and cover-slipped with neutral balsam immediately after air-drying.

For DHE staining, rabbits were anesthetized with isoflurane and then treated with enucleation of eye. The eyeball was dissected in oxygenated Ringer’s solution (pH 7.35; oxygenated with 95% O_2_ and 5% CO_2_) to keep cell viability. Samples were incubated with DHE solution (Beyotime, Shanghai, China, 5 μM/L, dissolved with PBS) in a light-protected chamber at 37°C for 40 min and immersed in 4% formaldehyde for 5 min. For the retina slice staining, 10, 20, and 30% sucrose were used to dehydrate the fixed retina. The retina was embedded in OCT compound (Sakura) and stored at −80°C. Fourteen-micrometer slices were cut (Leica CM 1950, Lecia, Germany) and washed three times for 15 min with 0.05 M TBS to wash away OCT. Slices were air-dried and mounted. The DHE images were obtained by fluorescence imaging microscope (Eclipse Ni, Nikon Inc, Japan).

### Pupillary Light Reflex

Rabbits were anesthetized by a mix of isoflurane and oxygen and followed with 30 min dim environment adaptation. Light stimuli provided by white LED was given to one eye and the pupil area was recorded with a near-infrared camera (JAI, Denmark). Each session was recorded for 30 s with a 10-Hz frame, and light stimulus were provided for 10–15 s when recording started for 5 s. Pupil area contraction percentage was calculated as (S0-Smin)/S0 × 100% (Smin: minimum pupil area during light stimulus; S0: pupil area during the dark environment).

### ERG Recording

After general anesthesia, compound tropicamide eye drops (Santen Pharmaceutical Co., LTD, Shiga Plant, Japan) were instilled in rabbits’ eyes to dilute the pupil, and 0.5% proparacaine hydrochloride eye drops (Alcon, Belgium) were used as corneal surface anesthesia. The circular corneal electrode was placed on the surface of the cornea of the rabbit, and the reference electrode of the silver needle was placed subcutaneously near the eye socket. The ground electrode of the silver needle was inserted subcutaneously into the back of the rabbit’s ear. ERG signals were amplified by an amplifier (Brownlee Precision Model 410, United States) at 128 Hz, and bandpass filtered between 1 and 1000 Hz. Light stimuli was applied by white LED and controlled by self-written Arduino code. Each session contains 10 stimuli, which lasts for 200 ms and is separated by 10 to 15 s randomly. The rabbit’s cornea was lubricated with 0.3% sodium hyaluronate eye drops (Santen Pharmaceutical Co., LTD, Shiga Plant, Japan) during recordings.

### Data Analysis

The pixel size of the spot diameter and retina thickness were measured in imaging-editing software (Adobe Photoshop CC 2018). The pixel-to-μm scale was obtained from the camera manufacturer’s software. For the length of spot diameter in the photocoagulation, eight more than clearly visible spots were chosen randomly. The maximum straight-line distance in each spot was manually marked, and the true distance was obtained according to the number of pixels. To calculate the thickness of the retina, two straight lines were manually marked at each region of photocoagulation and nonphotocoagulation, which were chosen randomly. Distance from the GCL to ONL in the nonphotocoagulation region was measured, and the distance from the GCL to the outer layer of the residual retina was measured in the photocoagulation region. The real thickness was estimated according to the mean number of pixels of the straight lines. The thickness of retina was normalized by the mean thickness of the nonphotocoagulation zone. Retinal thickness and spot diameter at each location were expressed as Mean ± Standard Error of Mean (SEM). The pupil area was measured by ImageJ (NIH, United States). Data were analyzed by GraphPad Prism software ver. 6.0c (GraphPad Software Inc., San Diego, CA, United States). *P*-values < .05 were considered statistically significant.

We used self-written python code to analyze the ERG data. The baseline of the ERG is the mean potential of the eye before stimuli during the dark adaption. The amplitude of a- and b-waves is measured from the baseline to the a-wave and the peak of a-wave to the peak of the b-wave, respectively. Each condition was repeated 10 times for each eye, and the average value was taken as the eye’s results. We calculated the average values of three eyes from two rabbits as the final results in this experiment.

## Data Availability Statement

The original contributions presented in the study are included in the article/supplementary material, further inquiries can be directed to the corresponding author/s.

## Ethics Statement

The animal study was reviewed and approved by Animal Care and Use Committee of Shanghai Medical College of Fudan University.

## Author Contributions

BY, AC, YY, and JZ conceived the experiments. ZW, CF, RY, YC, and TL conducted the experiments. TL, YC, AC, and YY participated in photocoagulation. CF did fundus photography and OCT scanning. ZW and RY conducted histologic analysis and pupillary light reflex test. ZW, RY, BY, and JZ wrote the manuscript. All authors contributed to the article and approved the submitted version.

## Conflict of Interest

The authors declare that the research was conducted in the absence of any commercial or financial relationships that could be construed as a potential conflict of interest.

## References

[B1] Ahn S. M. Ahn J. Cha S. Yun C. Park T. K. Goo Y. S. (2019). Development of a Post-vitrectomy Injection of N-methyl-N-nitrosourea as a Localized Retinal Degeneration Rabbit Model. *Exp Neurobiol* 28 62–73. 10.5607/en.2019.28.1.62 30853825 PMC6401555

[B2] Amirpour N. Karamali F. Rabiee F. Rezaei L. Esfandiari E. Razavi S. (2012). Differentiation of human embryonic stem cell-derived retinal progenitors into retinal cells by Sonic hedgehog and/or retinal pigmented epithelium and transplantation into the subretinal space of sodium iodate-injected rabbits. *Stem Cells Dev.* 21 42–53. 10.1089/scd.2011.0073 21456900

[B3] Blumenkranz M. S. Yellachich D. Andersen D. E. Wiltberger M. W. Mordaunt D. Marcellino G. R. (2006). Semiautomated patterned scanning laser for retinal photocoagulation. *Retina* 26 370–376. 10.1097/00006982-200603000-00024 16508446

[B4] Chhablani J. Roh Y. J. Jobling A. I. Fletcher E. L. Lek J. J. Bansal P. (2018). Restorative retinal laser therapy: present state and future directions. *Surv. Ophthalmol.* 63 307–328. 10.1016/j.survophthal.2017.09.008 28987614

[B5] Fong D. S. Girach A. Boney A. (2007). Visual side effects of successful scatter laser photocoagulation surgery for proliferative diabetic retinopathy: a literature review. *Retina* 27 816–824. 10.1097/IAE.0b013e318042d32c 17891003

[B6] Framme C. Alt C. Schnell S. Sherwood M. Brinkmann R. Lin C. P. (2007). Selective targeting of the retinal pigment epithelium in rabbit eyes with a scanning laser beam. *Invest Ophthalmol Vis Sci* 48 1782–1792. 10.1167/iovs.06-0797 17389512

[B7] Framme C. Schuele G. Roider J. Birngruber R. Brinkmann R. (2004). Influence of pulse duration and pulse number in selective RPE laser treatment. *Lasers. Surg. Med.* 34 206–215. 10.1002/lsm.20022 15022247

[B8] Isago H. Sugano E. Murayama N. Tamai M. Tomita H. (2013). Establishment of monocular-limited photoreceptor degeneration models in rabbits. *BMC Ophthalmol.* 13:19. 10.1186/1471-2415-13-19 23683117 PMC3679785

[B9] Jain A. Blumenkranz M. S. Paulus Y. Wiltberger M. W. Andersen D. E. Huie P. (2008). Effect of pulse duration on size and character of the lesion in retinal photocoagulation. *Arch Ophthalmol* 126 78–85. 10.1001/archophthalmol.2007.29 18195222

[B10] Jang S. Y. Cho I. H. Yang J. Y. Park H. Y. Woo S. E. Madrakhimov S. B. (2019). The retinal pigment epithelial response after retinal laser photocoagulation in diabetic mice. *Lasers Med Sci* 34 179–190. 10.1007/s10103-018-2680-9 30499004

[B11] Kern K. Mertineit C. L. Brinkmann R. Miura Y. (2018). Expression of heat shock protein 70 and cell death kinetics after different thermal impacts on cultured retinal pigment epithelial cells. *Exp. Eye Res.* 170 117–126. 10.1016/j.exer.2018.02.013 29454858

[B12] Kondo M. Sakai T. Komeima K. Kurimoto Y. Ueno S. Nishizawa Y. (2009). Generation of a transgenic rabbit model of retinal degeneration. *Invest Ophthalmol Vis Sci* 50 1371–1377. 10.1167/iovs.08-2863 19074802

[B13] Liang L. Katagiri Y. Franco L. M. Yamauchi Y. Enzmann V. Kaplan H. J. (2008). Long-term cellular and regional specificity of the photoreceptor toxin, iodoacetic acid (IAA), in the rabbit retina. *Vis Neurosci* 25 167–177. 10.1017/S0952523808080401 18442439

[B14] Liu G. Chen L. Cai Q. Wu H. Chen Z. Zhang X. (2018). Streptozotocininduced diabetic mice exhibit reduced experimental choroidal neovascularization but not corneal neovascularization. *Mol Med Rep* 18 4388–4398. 10.3892/mmr.2018.9445 30221697 PMC6172380

[B15] Lock J. H. Fong K. C. (2011). An update on retinal laser therapy. *Clin Exp Optom* 94 43–51. 10.1111/j.1444-0938.2010.00529.x 21039846

[B16] Matsumoto M. Miki T. Obana A. Shiraki K. Suh J. H. (1992). Indocyanine green enhanced photocoagulation in the pigmented rabbit. *Nippon Ganka Gakkai Zasshi* 96 742–748. 1626476

[B17] McHugh D. England C. van der Zypen E. Marshall J. Fankhauser F. Fankhauser-Kwasnieska S. (1995). Irradiation of rabbit retina with diode and Nd:YAG lasers. *Br J Ophthalmol* 79 672–677. 10.1136/bjo.79.7.672 7662634 PMC505197

[B18] Menon I. A. Wakeham D. C. Persad S. D. Avaria M. Trope G. E. Basu P. K. (1992). Quantitative determination of the melanin contents in ocular tissues from human blue and brown eyes. *J Ocul Pharmacol* 8 35–42. 10.1089/jop.1992.8.35 1402293

[B19] Nishida K. Kamei M. Kondo M. Sakaguchi H. Suzuki M. Fujikado T. (2010). Efficacy of suprachoroidal-transretinal stimulation in a rabbit model of retinal degeneration. *Invest. Ophthalmol. Vis. Sci.* 51 2263–2268. 10.1167/iovs.09-4120 19933186

[B20] Obana A. Miki T. (1989). The effect of melanin and hemoglobin on the dye laser photocoagulation in pigmented and albino rabbits. *Nippon Ganka Gakkai Zasshi* 93 844–851. 2610165

[B21] Pender P. M. Benson W. E. Compton H. Cox G. B. (1981). The effects of panretinal photocoagulation on dark adaptation in diabetics with proliferative retinopathy. *Ophthalmology* 88 635–638. 10.1016/s0161-6420(81)34977-x 7196565

[B22] Pennesi M. E. Neuringer M. Courtney R. J. (2012). Animal models of age related macular degeneration. *Mol. Aspects Med.* 33 487–509. 10.1016/j.mam.2012.06.003 22705444 PMC3770531

[B23] Petrus-Reurer S. Bartuma H. Aronsson M. Westman S. Lanner F. André H. (2017). Integration of subretinal suspension transplants of human embryonic stem cell-derived retinal pigment epithelial cells in a large-eyed model of geographic atrophy. *Invest. Ophthalmol. Vis. Sci.* 58 1314–1322. 10.1167/iovs.16-20738 28241319

[B24] Petrus-Reurer S. Bartuma H. Aronsson M. Westman S. Lanner F. Kvanta A. (2018). Subretinal transplantation of human embryonic stem cell derived-retinal pigment epithelial cells into a large-eyed model of geographic atrophy. *J. Vis. Exp.* 131:56702. 10.3791/56702 29443034 PMC5908677

[B25] Plaza Reyes A. Petrus-Reurer S. Antonsson L. Stenfelt S. Bartuma H. Panula S. (2016). Xeno-free and defined human embryonic stem cell-derived retinal pigment epithelial cells functionally integrate in a large-eyed preclinical model. *Stem Cell Rep.* 6 9–17. 10.1016/j.stemcr.2015.11.008 26724907 PMC4720022

[B26] Querques G. Cicinelli M. V. Rabiolo A. de Vitis L. Sacconi R. Querques L. (2018). Laser photocoagulation as treatment of non-exudative age-related macular degeneration: state-of-the-art and future perspectives. *Graefes Arch Clin Exp Ophthalmol* 256 1–9. 10.1007/s00417-017-3848-x 29177712

[B27] Reddy S. V. Husain D. (2018). Panretinal Photocoagulation: A Review of Complications. *Semin Ophthalmol* 33 83–88. 10.1080/08820538.2017.1353820 29172937

[B28] Saenz-de-Viteri M. Heras-Mulero H. Fernández-Robredo P. Recalde S. Hernández M. Reiter N. (2014). Oxidative stress and histological changes in a model of retinal phototoxicity in rabbits. *Oxid Med Cell Longev* 2014 637137. 10.1155/2014/637137 24991304 PMC4058492

[B29] Sasaki M. Ozawa Y. Kurihara T. Kubota S. Yuki K. Noda K. (2010). Neurodegenerative influence of oxidative stress in the retina of a murine model of diabetes. *Diabetologia* 53 971–979. 10.1007/s00125-009-1655-6 20162412 PMC2850533

[B30] Schmidt S. Y. Peisch R. D. (1986). Melanin concentration in normal human retinal pigment epithelium. Regional variation and age-related reduction. *Invest Ophthalmol Vis Sci* 27 1063–1067. 3721785

[B31] Schraermeyer U. Heimann K. (1999). Current understanding on the role of retinal pigment epithelium and its pigmentation. *Pigment Cell Res* 12 219–236. 10.1111/j.1600-0749.1999.tb00755.x 10454290

[B32] Schubert H. D. Federman J. L. (1989). A comparison of CW Nd:YAG contact transscleral cyclophotocoagulation with cyclocryopexy. *Invest Ophthalmol Vis Sci* 30 536–542. 2925323

[B33] Shirai H. Mandai M. Matsushita K. Kuwahara A. Yonemura S. Nakano T. (2016). Transplantation of human embryonic stem cell-derived retinal tissue in two primate models of retinal degeneration. *Proc. Natl. Acad. Sci. U. S. A.* 113 E81–E90. 10.1073/pnas.1512590113 26699487 PMC4711854

[B34] Suh J. H. Miki T. Obana A. Shiraki K. Matsumoto M. (1991). Effects of indocyanine green dye enhanced diode laser photocoagulation in non-pigmented rabbit eyes. *Osaka City Med. J* 37 89–106. 1792069

[B35] Weiter J. J. Delori F. C. Wing G. L. Fitch K. A. (1986). Retinal pigment epithelial lipofuscin and melanin and choroidal melanin in human eyes. *Invest. Ophthalmol. Vis. Sci.* 27 145–152. 3943941

[B36] Yamauchi Y. Agawa T. Tsukahara R. Kimura K. Yamakawa N. Miura M. (2011). Correlation between high-resolution optical coherence tomography (OCT) images and histopathology in an iodoacetic acid-induced model of retinal degeneration in rabbits. *Br J Ophthalmol* 95 1157–1160. 10.1136/bjo.2010.186718 21030415

