# Corrigendum: Large-Area Photoreceptor Degeneration Model in Rabbits by Photocoagulation and Oxidative Stress in the Retina

**DOI:** 10.3389/fnins.2021.738004

**Published:** 2021-07-26

**Authors:** Zhexuan Wang, Chenli Feng, Ruyi Yang, Tingting Liu, Yin Chen, Aihua Chen, Biao Yan, Yuanzhi Yuan, Jiayi Zhang

**Affiliations:** ^1^State Key Laboratory of Medical Neurobiology, Department of Ophthalmology, MOE Frontiers Center for Brain Science, Zhongshan Hospital, Institutes for Brain Science, Fudan University, Shanghai, China; ^2^Department of Ophthalmology, Eye and Ent Hospital of Fudan University, Shanghai, China; ^3^Key Laboratory of Brain Functional Genomics, Primate Research Center, East China Normal University, Shanghai, China

**Keywords:** photocoagulation, light pupillary reflex, rabbit model, oxidative stress, electroretinography

In the original article, we neglected to include the funder **Research and Development Fund of Zhongshan Hospital**, **2020ZSFZ19** to **Chenli Feng**.

In the published article, there was an error in affiliation **label for Zhexuan Wang, Chenli Feng and Ruyi Yang**. Instead of “**Zhexuan Wang**^**1,2**^^**†**^**, Chenli Feng**^**1,2**^^**†**^**, Ruyi Yang**^**1,2**^^**†**^”, it should be “**Zhexuan Wang**^**1**^^**†**^**, Chenli Feng**^**1**^^**†**^**, Ruyi Yang**^**1**^^**†**^”.

The authors apologize for this error and state that this does not change the scientific conclusions of the article in any way. The original article has been updated.

## Publisher's Note

All claims expressed in this article are solely those of the authors and do not necessarily represent those of their affiliated organizations, or those of the publisher, the editors and the reviewers. Any product that may be evaluated in this article, or claim that may be made by its manufacturer, is not guaranteed or endorsed by the publisher.

